# Looking beyond the smokescreen: can the oral microbiome be a tool or target in the management of tobacco-associated oral cancer?

**DOI:** 10.3332/ecancer.2021.1179

**Published:** 2021-02-01

**Authors:** Snehal Kadam, Madhusoodhanan Vandana, Sudhanshu Patwardhan, Karishma S Kaushik

**Affiliations:** 1 Human-Relevant Infection Biology Group, Institute of Bioinformatics and Biotechnology, Savitribai Phule Pune University, Pune 411007, India; 2 Centre for Health Research and Education, University of Southampton Science Park, Chilworth, Hampshire SO16 7NP, UK

**Keywords:** oral microbiome, biofilms, tobacco, potentially malignant lesions, oral cancer

## Abstract

A wide range of microbes inhabit the oral cavity, and bacterial and fungal communities most often exist as structured communities or biofilms. The use of tobacco alters the structure of the oral microbiome, including that of potentially malignant lesions, and the altered oral microbiome influences key microenvironmental changes such as chronic inflammation, secretion of carcinogenic toxins, cellular and tissue remodelling and suppression of apoptosis. Given this, it is clear that the bacterial and fungal biofilms in potentially malignant states are likely not passive entities, but could play a critical role in shaping potential malignant and carcinogenic conditions. This holds potential towards leveraging the oral microbiome for the management of tobacco-associated potentially malignant lesions and oral cancer. Here, we explore this line of investigation by reviewing the effects of tobacco in shaping the oral microbiome, and analyse the available evidence in the light of the microbiome of oral potentially malignant and cancerous lesions, and the role of dysbiosis in carcinogenesis. Finally, we discuss possible interventions and approaches using which the oral microbiome could be leveraged towards precision-based oral cancer therapeutics.

## Tobacco use and oral cancer

Tobacco-associated disease is a global public health threat [1]. There are ~1.3 billion users of tobacco worldwide [2], of which an overwhelming majority (~80%) live in low- and middle-income countries. While a large proportion of tobacco users worldwide are cigarette smokers (~1 billion), the use of smokeless tobacco forms (nearly 30% in South and South-East Asia [3]) is also a grave concern. In India alone, there are ~300 million consumers of smokeless tobacco forms, and it is the most prevalent form of tobacco use among women in the country (India Report from CHRE [4]). Tobacco use is associated with a range of adverse health consequences [5, 6], notably the development of oral cancer [7].

Globally, tobacco use is responsible for 25% of all cancer-related deaths (2.4 million deaths from tobacco-use associated cancers) [8]. Cigarette smoke contains approximately 7,000 chemicals, of which at least 70 are known carcinogens [9–11]. Smoking-associated oral malignant changes are typically seen in the labial or buccal mucosa, tongue, gingiva, palate, alveolar mucosa, lips and salivary glands [12–15]. On the other hand, smokeless tobacco forms are chewed, placed in close contact with the oral mucosa or applied over the teeth and gums. Smokeless tobacco is consumed as raw leaves (local names such as *khaini, misri, gutka, zarda, toombak*) or dissolvable forms (lozenges, sticks, strips) [3, 16], often containing additives such as areca nut, betel quid, catechu, slaked lime, ash, sodium bicarbonate, as well as flavouring agents such as menthol and plant oils. The process of tobacco curing, fermentation and ageing, results in the production of tobacco-specific nitrosamines (TSNAs), which are the major group of carcinogens in smokeless tobacco [17–19].

## Current management of tobacco-associated premalignant changes and oral cancer

Tobacco-associated oral cancer typically starts as premalignant lesions, which through a series of genetic and molecular changes, influenced by oral microenvironmental factors [20, 21], undergoes malignant transformation to oral cancer. The most common form of oral cancer squamous cell carcinoma [22], which most often develops from potentially malignant lesions such as oral submucous fibrosis, leukoplakia, erythroplakia, among others [23, 24].

Currently, the mainstay of management for oral premalignant lesions is observation with frequent clinical examinations, along with education towards cessation. This is not only a passive and suboptimal approach, but is also limited by access to healthcare and success of cessation efforts. On the other hand, active interventional approaches include surgical resection and ablation. These are limited by ‘field effects’ where potentially malignant changes, though not phenotypically visible, extend to larger areas beyond the lesion, as well as severe disfigurement and impairment of function. Finally, limited medical approaches such as retinoic acid, Vitamin E, and natural compounds are either limited by toxicity (retinoic acid) or lack of thorough clinical evaluations [20]. As evident, there is an impasse in the management of tobacco-associated potentially malignant lesions, and current management strategies fail to account for the complexity of the disease state.

## Tobacco, the oral microbiome and oral cancer

Tobacco-associated potentially malignant lesions exist and develop in a dynamic oral microenvironment, that includes diverse, multi-species microbial communities [25–28], most often observed as biofilms. It is widely recognised that distinct microbial signatures influence key processes such as chronic inflammation and carcinogenesis [29, 30]. This offers the exciting possibility that the oral microbiome (or biofilms) can be a tool or target in the management of tobacco-associated potentially malignant lesions. Here, we explore this line of investigation by reviewing the increasingly recognized role of tobacco in shaping the oral microbiome, focusing on the bacterial and fungal communities (Table 1). We then look at the effects of bacterial and fungal dysbiosis on key processes in oral potentially malignant and cancerous lesions. Finally, we discuss possible interventions and approaches with which the oral microbiome can be leveraged towards precision-based oral cancer therapeutics.

### How tobacco affects the oral microbiome

Tobacco is known to influence the inflammatory response of the oral cavity [31–42]. Overall, this contributes to a pro-inflammatory microenvironment eventually leading to chronic inflammation, which is known to be associated with oral carcinogenesis [43–47]. However, it is evident that tobacco exposure (in various forms) not only results in carcinogenic changes in the oral mucosa [48–57], but also shapes the oral microbiome. Several studies have characterized the oral microbiome in tobacco users [36, 58–64], using samples such as tumour or lesion biopsies, oral biofilm swabs, oral cavity rinses or saliva, from a diverse range of tobacco users.

#### Alteration in bacterial species profiles

In one study, tobacco smokers and non-smokers were sampled for oral marginal and subgingival plaques [36]. During this study, all subjects received oral prophylaxis to remove any initial plaque and were asked to cover six teeth while brushing to avoid loss of the plaque. After 24 hours, the plaque samples were collected and the procedure was repeated to obtain samples at different time points up to 7 days. This model allowed the study of undisturbed plaque from subjects for the given time periods. About 71% of the oral plaque community in non-smokers remained stable over the 7-day period, whereas this number was only 46% in smokers, indicating greater fluctuations in smoker plaques. Over a period of 7 days, smokers acquired species like *Lactobacillus, Fusobacterium, Centipeda periodontii, Pseudomonas, Treponema, Leptotrichia, Synergistes, Propionibacterium* and *Cardiobacterium*, which were absent in non-smokers [36]. In a similar study, tobacco smoking contributed to a pathogen-rich environment as seen by the presence of species such as *F. nucleatum, F. naviforme, A. johnsonii, A. baumannii, A. haemolyticus, S. mutans*, and low abundance of *Streptococcus sanguinis, S. oralis, Actinomyces viscosus, A. israelii, A. dentalis, Neisseria subflava* [63, 64].

In another study of smokers and non-smokers, with further subgroups of treated and non-treated for periodontitis, *Fusobacterium* was found to be higher in both untreated and treated smoker groups [65]. Additionally, the same study found *Bacteroides forsythus, Peptostreptococcus micros* and *Campylobacter rectus*, along with *Fusobacterium nucleatum*, to be the most prominent microbial signature for smokers. Though studies have noted an increase in *Fusobacteria* in smoker populations, some studies report an opposite trend [59, 66, 67]. Interestingly, under *in vitro* conditions, smokeless tobacco aqueous extracts and TSNAs demonstrated either a reduction of growth for *Fusobacterium nucleatum* or no change in profile. All smokeless extracts promoted the growth of *Peptostreptococcus micros, Streptococcus anginosus, S. constellatus, S. sanguinis* and *Veillonella parvula* [68]*.* For certain species, this also correlates to what is seen *in vivo*, where *Streptococcus anginosus* was significantly higher in chewers having oral lesions [69], and has been identified in various head and neck cancers [70–72]. Notably, *Streptococcus infantis* was more abundant in current chewers compared to past users or those who never used the tobacco product [69], indicating that some differences in microbiome due to betel quid chewing could be reversible.

On the other hand, clinical saliva samples of tobacco smokers revealed reduced diversity of Gram-positive bacteria compared to non-smokers, with only nine different species cultured from smokers compared to eighteen different species from non-smokers [61]. Certain *Proteobacteria* were also reduced in smokers [59, 60, 62].

#### Alteration in bacterial gene expression profiles

In addition to altered species profiles in tobacco users, tobacco smoke exposure elicits different gene expression profiles in multi-species pathogenic and commensal biofilms [35]. Commensal biofilms were grown by seeding *Streptococcus oralis, Streptococcus sanguis, Streptococcus mitis, Veillonella parvula, Neisseria mucosa,* and *Actinomyces naeslundii* in a mixture of Brain -Heart Infusion broth (BHI) and artificial saliva (1:1). Pathogenic biofilms were grown by seeding commensals along with *F. nucleatum*, and incubating for 24 hours, followed by further seeding of *Porphyromonas gingivalis, Filifactor alocis, Dialister pneumosintes, Selenonomas sputigena, Selenominas noxia, Catonella morbi, Parvimonas micra and Tannerella forsythia*. Smoke exposure upregulated fermentative pathways in both pathogenic and commensal biofilms. Fermentative pathways among several bacteria can lead to the formation of metabolites like butyrate [73], and the role of butyrate has been studied in various cancers [74, 75]. Commensal biofilms in tobacco smoke environments faced a loss of viability and elicited an early proinflammatory immune response (seen as an increase in various interleukins and macrophage inflammatory proteins) from immortalised human oral keratinocytes (OKF6/TERT-2) [35]. Pathogenic biofilms, however, did not show any significant loss of viability during the same time period, but did elicit a late immune response. This could indicate that tobacco exposure leads to elimination of commensal biofilms, while selecting for pathogenic biofilms. This transition from a commensal dominant to a pathogen dominant biofilm with tobacco usage could serve as a marker for oral health decline and increased risks of lesion progression.

#### Effect on bacterial interspecies interactions

When *Porphyromonas gingivalis* grown in cigarette smoke extract-conditioned media (compared to non-conditioned media) was added on top of *Streptococcus gordonii* on saliva-coated coverslips in flow cells, a three-fold increase in the dual-species biofilm height and two-fold increase in total microcolony numbers were detected [76]. Peripheral blood mononuclear cells challenged with cigarette smoke extract-exposed* P. gingivalis* biofilms (on pellicle coated discs) exhibited lower Interleukin (IL)-6 and Tumor Necrosis Factor (TNF)-α, compared to control biofilms. Cigarette smoke extract exposure was thus seen to promote dual-species biofilms of *P. gingivalis* and *S. gordonii,* and lowers the proinflammatory response elicited by *P. gingivalis*. Cigarette smoke extract-exposure increased *P. gingivalis* binding to immobilised Glyceraldehyde 3-phosphate dehydrogenase (GAPDH), a FimA ligand on *S. gordonii*. a FimA ligand on *S. gordonii*. Additionally, cigarette smoke extract-exposure was shown to increase FimA protein levels, an important virulence factor that helps in adhesion and attachment, and also increases biofilm formation by* P. gingivalis* [77]. A recent study has shown that *P. gingivalis* invaded OKF6/TERT cells less in the presence of commensals such as *S. gordonii* [78]. An increase in dual-biofilm formation upon cigarette smoke extract-exposure suggests that tobacco could possibly alter the interactions between the two bacterial species allowing pathogens to use existing commensals to establish themselves in the oral cavity. Various studies have isolated *P. gingivalis* from oral tumours [79, 80], and infection with *P. gingivalis* resulted in increased tongue lesion size in a 4-nitroquinoline 1-oxide (4NQO) based cancer mouse model [81]. Moreover, infection with *P. gingivalis* along with 4-quinoline oxide (QO), a carcinogen, led 65% of the mice to develop squamous cell carcinoma compared to 45% in mice treated with only 4NQO, indicating that *P. gingivalis* enhanced carcinogenesis. *P. gingivalis* also showed increased staining in gingival carcinomas as compared to *S. gordonii* [80], supporting the non-invasiveness of *S. gordonii* with respect to gingival epithelial cells* in vitro* [82].

#### Effect on fungal species profiles

Among fungal species, *Candida albicans* and non-albicans *Candida* species such as *C. tropicalis, C. krusei, C. glabrata* have been isolated from various oral lesions [83–87]. *C. albicans* was significantly higher in oral swabs of waterpipe smokers compared to non-smokers [66]. Saliva samples from tobacco users had higher levels of *Candida albicans* and *C. tropicalis* and *C. krusei* was unchanged compared to non-tobacco users [88]. This trend was also observed with smokeless tobacco users (*gutka* and betel-quid chewers), who had a higher *Candida* carriage, compared to non-chewers, with *C. albicans* being the most commonly present species [89].

Different concentrations of cigarette smoke condensate have been shown to result in increased adhesion on glass slides as well as increased metabolic activity in cultures of *C. albicans* [90]. *C. albicans* also formed more biofilms on collagen scaffolds, as seen by scanning electron microscopy as well as crystal violet staining. This increased adhesion and biofilm formation of *Candida* biofilms in presence of cigarette smoke condensate has also been noted for orthodontic materials like acrylic resin and bands [91]. Cigarette smoke condensate (CSC), in the presence of 10% serum at 37°C (hyphae-forming conditions), was observed to increase *C. albicans in vitro* hyphal formation and chitin production [92]*. C. albicans* pretreated with CSC were not only able to adhere more to human gingival fibroblasts, but also proliferated more within 48 hours. A higher number of CSC-pretreated *Candida* cells transitioned to hyphal form upon contact with the fibroblasts under hyphal specific conditions compared to those not treated with CSC. Attachment to host cells and increased hyphal formation are important for the invasiveness of *Candida* [93], with *Candida* species isolated from oral mucosal lesions associated with tobacco use were found to be in their hyphal forms [85].

So far, the evidence strongly establishes that tobacco use alters the distribution, diversity and abundance of oral bacterial and fungal species, relative to non-users. The fact that the studies vary in their findings, underscores that various factors such as tobacco type, usage pattern, duration and frequency, underlying predisposing factors, health conditions and diet, play an additional role [94]. Notwithstanding this, altered oral microbial communities resulting from tobacco use are very likely to shape the microbiome of tobacco-associated potentially malignant lesions.

### Microbes associated with oral potentially malignant lesions and cancer

Microbes, often as biofilms, have been found in close association with potentially malignant lesions and Oral Squamous Cell Carcinoma (OSCC) tumours in the oral cavity [86, 95–101]. Notably, the abundance, distribution, diversity have been observed to vary not only between tobacco-users and non-users, but also across tumour and non-tumour sites in OSCC patients [98, 102, 103]. Bacterial communities on the surface of oral carcinomas in tobacco-users have to be enriched for *Fusobacterium, Actinomyces, Clostridium, Enterobacteriaceae* and *Streptococcus* species, *Klebsiella pneumoniae*, *Enterococcus faecalis, Veillonella, Prevotella, Porphyromonas* and *Clostridium* [98, 102, 103]. In one study, three groups, comprising of OSCC tumour patients, high-risk individuals (smokers and alcohol users without signs of OSCC) and healthy individuals, were analysed for microbial species and abundance (either at tumour sites of OSCC subjects or normal mucosa in high-risk and healthy subjects) [100]. Patients with OSCC tumours showed a higher presence of anaerobes, relative to the other two groups, with *Fusobacterium* and *Prevotella* seen to predominate. Another study identified a large cohort of bacteria within OSCC tissues, as visualised by fluorescent in situ hybridisation [96]. Bacteria were found to be present throughout the OSCC tissue. Bacterial identification via 16S rRNA sequencing revealed that *Clavibacter michiganensis subsp. tessellarius, Fusobacterium naviforme* and *Ralstonia insidiosa* were at least 30% more in OSCC samples. *Streptococcus anginosus* was also identified in a subset of patient in this study. Notably, *Streptococcus anginosus* has been found in close association with many head and neck carcinomas [70, 71]. Notably, *S. anginosus* isolated from OSCC samples was genotypically identical to* S. anginosus* isolated from dental plaques of the same patients, and was not detected in saliva samples, indicating that plaques could be locations where the bacteria accumulate in the oral cavity [71].

*Candida* species have been isolated from patients having malignant oral squamous cell carcinoma, atypical lichen planus and chronic candidiasis, and *Candida* species from dorsum tongue of asymptomatic carriers with healthy mucosa were used as controls [83]. *Candida albicans* was the most commonly occurring species in all groups. *Candida albicans* strains of different biotypes isolated from leukoplakia and erythroleukoplakia have demonstrated high nitrosation potential, quantified as the ability to form N-nitroso benzylmethylamine from N-benzylmethylamine and nitrite [104]. Higher nitrosation potential isolates correlated with advanced stages of lesions. Other *Candida* species, such as *C. tropicalis* and *C. parapsilosis*, isolated from these lesions ranked lower in their nitrosation potential. The ability to produce carcinogens like nitrosamines has been suggested to contribute to malignant transformation in the oral cavity [105–107]. Further, *in* vitro analysis revealed that isolates from the carcinoma and candidiasis groups were able to form significantly more biofilms as quantified by metabolic activity (XTT); notably, *Candida albicans* formed less biofilms compared to other strains. On the other hand, no difference was found in lipolytic activity, proteolytic activity and hydrophobicity. Though the virulence factors had differential levels in different clinical conditions, there was no association of virulence factors with individual strain types. This indicates that *Candida* pathogenesis is likely to be influenced by an interplay of multiple factors like virulence, abundance of species, as well as other underlying factors like the immune state of the patient and co-infecting species.

In a recent study, the diversity and distribution of microbial signatures was largely seen to reduce with cancer progression. When samples of normal, epithelial precursor lesions (dysplasia, hyperplasia or hyperkeratosis) and OSCC individuals were analysed for microbial composition, a multidimensional scaling plot revealed that the distribution was more condensed for OSCC patients than for the other two groups [103]. Harvesting of tissue samples from oral tumours and 16S rRNA sequencing based identification of microbial isolates revealed that the microbiome of superficial tissues of the tumour housed additional species which were absent in the deep tumour tissues [108]. This indicates a specialisation within the tumour itself, where not all species in the oral cavity would become associated with and survive in the tumour microenvironment.

Taken together, certain key microbial players are observed to be consistently detected in association with oral potentially malignant lesions and OSCC, including *Fusobacterium, Candida, Porphyromonas, Streptococcus*, *Veillonella* and *Prevotella*. While evidence is limited in this regard, it is likely that the microbial profile changes with lesion progression, pointing to the imminent role of the microbiome in shaping inflammatory and carcinogenic processes.

### Oral dysbiosis and cancer: how could the altered oral microbiome play a role in carcinogenesis?

Microbial dysbiosis is well-known to be associated with carcinogenic processes [109–112], either via inflammatory changes, or the production of toxins or metabolites. It is likely that these processes play out in potentially malignant and OSCC lesions, and could initiate and promote a series of inflammatory and potentially carcinogenic changes in the oral cavity. In general, multiple oral species, via secreted endotoxins and metabolic byproducts, induce production of pro-inflammatory cytokines (such as TNFɑ, IL-1, IL-6, IL-8), other immune signaling factors (such as Matrix Metallo-Proteinases (MMPs), Granulocye-Macrophage Colony-Stimulating Factor (GM-CSF)), degradation of tissues, inhibition of antibacterial activities of immune cells and invasion of host tissues [26, 113–116]. This results in a chronic inflammatory microenvironment, known to affect key carcinogenic processes such as cell growth, proliferation and migration, apoptosis and differentiation to tumour-like phenotypes [105, 113, 117–119]. In the context of oral carcinogenesis, notable pathogens include *Fusobacterium nucleatum*, *Porphyromonas gingivalis* and *Candida* species.

*Porphyromonas gingivalis* and *Fusobacterium nucleatum* produce sulphur compounds that induce cell proliferation, migration, invasion and tumour angiogenesis. *In vitro* infection of an oral cancerous cell line (OQ01) with *Fusobacterium nucleatum* showed increased IL-8 production and invasiveness of the cell line [120]. In another *in vitro* study, human OSCC cells showed increased proliferation and invasiveness when co-incubated with *P. gingivalis* and* F. nucleatum*. Prolonged exposure resulted in a change in OSCC morphology from polygonal to elongated, a decrease in the levels of the epithelial marker Cytokeratin-13 and an increase of mesenchymal markers (N-cadherin and α-Smooth Muscle Acton (SMA)), indicating an Epithelial-to-Mesemchymal Transition (EMT) phenotype [121]. *P. gingivalis* has also been shown to inhibit apoptosis, and enhance cell proliferation and cellular invasion with *F. nucleatum* [122–129]*,* and their role in promoting OSCC has been characterised extensively [26, 99, 130]. In an *in vivo* 4NQO-induced carcinoma model, mice administered with 4NQO and co-infected with both *P. gingivalis* and *F. nucleatum* developed tongue tumours 2.5 times in size compared to mice only administered 4NQO and had an increased expression of the oncogene cyclin D1 [131]*.*

*Candida* species have been widely reported in association with oral potentially malignant lesions and in OSCC samples. Through hyphal formation, that enables active penetration or endocytosis,* Candida* species have been shown to invade epithelial cells [132–136]. Further contact-sensing and hyphal extension, combined with secretion of toxins and virulence factors induces damage to the oral epithelia [137–139]. *Candida* species also produce nitrosamines and their nitrosation potential can lead to DNA damage, and eventually initiate carcinogenic changes [107, 140]. *Candida* species have been isolated from lesions of oral leukoplakia, where a higher nitrosation potential of the fungal strain was associated with an advanced potentially malignant stage [104]. Therefore, it is likely that increased *Candida* abundance, adhesion and invasion with tobacco usage, and the potential of *Candida*-induced nitrosative changes plays a role in the development of potentially malignant lesions and progression to cancer [139, 141].

### Leveraging the oral microbiome as a tool or target in the management of tobacco-associated oral potentially malignant lesions and cancer

**Figure 1.**
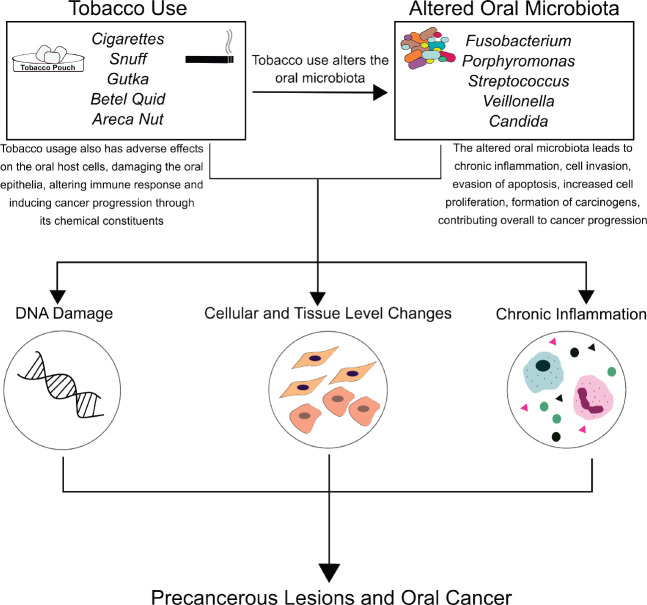
Role of tobacco and microbes in oral carcinogenesis: Tobacco use, its forms, products and additives (cigarette, snuff, *gutka*, betel quid, areca nut), and derivatives (like tobacco-specific nitrosamines) have direct carcinogenic effects on the oral mucosa. Tobacco use also alters the oral microbiome, resulting in distinct microbial signatures in normal mucosa, potentially malignant lesions and cancerous tissue. Together, this altered microbiome and tobacco use result in a proinflammatory microenvironment, contributing to potentially malignant lesions, and oral carcinogenesis.

Taken together, the use of tobacco alters the oral microbiome, and the oral microbiome influences key pathways involved in inflammation and carcinogenesis (Figure 1). Given this, it is clear that the oral microbiome in potentially malignant states plays a critical role in shaping the oral carcinogenic microenvironment. Therefore, leveraging the oral microbiome towards the management of tobacco-associated potentially malignant lesions and oral cancer could hold potential. For this, there are several strategies and approaches that could be adopted.

*Profiling the oral microbiome in tobacco-associated potentially malignant lesions:* Using a combination of standard microbiology approaches and molecular tools, the biogeography of the oral microbiome in a range of tobacco-associated clinical conditions, across variations in diet, lifestyle, associated risk factors, can be profiled. This will serve as a baseline for screening and interventional tools in local populations, and is particularly important given the variations in the microbial profile across study conditions.

*Probing the microbiome of tobacco-associated potentially malignant lesions as a cancer screening tool:* Based on previous studies, the progression of potentially malignant lesions to oral cancer is associated with an alteration in the microbiome, and levels of microbial metabolites. Detection of these ‘signature’ markers in potentially malignant lesions could be leveraged as screening approaches. This would serve as a valuable addition to macroscopic (visual) screening for cancerous changes, and be less invasive as compared with routine biopsies. Most importantly, identifying microbial changes early in cancer progression would provide the possibility of targeting the oral microbiome, an active interventional approach to preventing progression to oral cancer.

*Targeting the oral microbiome in the management of potentially malignant lesions and oral cancer:* For this, a wide range of antimicrobial approaches, including conventional antibiotics and antifungal agents, can be employed. However, given the rise in antibiotic resistance, novel antimicrobial approaches such as peptides, natural extracts, polyphenols, nucleic-acid mimics and structural analogues can be evaluated for antimicrobial and antibiofilm effects. Additional approaches could include targeting the proinflammatory markers, including cytokines and growth factors, known to be dysregulated in potentially malignant lesions, and cancer progression states.

## Conclusion

In conclusion, exploring interactions between tobacco use, the oral microbiome and carcinogenesis could not only lead to identification of potential biomarkers, but also lead to precision-treatment approaches that shape the oral microbiome towards a better therapeutic outcomes for oral potentially malignant lesions and cancer. This will provide comprehensive insights into the ‘microbiome paradigm’ for the management of tobacco-associated oral carcinogenesis, and open unexplored lines of investigation into precision-based oral cancer therapeutics.

## Conflicts of interest

Dr Sudhanshu Patwardhan (SP) is a paid Director for Centre for Health Research and Education (CHRE) UK, an independent healthcare company, which works on projects on smoking cessation and cancer prevention globally. CHRE has received grants from Foundation for a Smoke-free World, Inc for some of its smoking cessation projects. For this article, SP or CHRE did not receive any external funding.

## Funding statement

The academic appointments of Karishma S Kaushik (KSK) and Snehal Kadam are funded via the Ramalingaswami Re-entry Fellowship (to KSK), Department of Biotechnology, Government of India. This review was commissioned and funded by CHRE.

## Figures and Tables

**Table 1.** Tobacco-associated oral microbiome changes associated with the development and progression of premalignant lesions and oral cancer.

	Tobacco components associated with the condition	Microbes associated with the condition	Reference
Early inflammation to chronic inflammation	Smokeless forms: Betel (Areca) nut, Gutka, Iqmik Smoking forms: Nicotine, cigarette smoke	Reduction in commensal microflora while no loss in pathogenic forms. *Bacterial—Porphyromonas gingivalis, Streptococcus anginosus* *Fungal—Candida albicans*	[31, 35, 40, 55, 79, 142–144]
Leukoplakia	Smokeless form: Betel (Areca) nut, Toombak Smoking form: Cigarette smoke	Bacterial–Streptococcus anginosus, *Oribacterium*, *S*treptococcus *infantis, Actinomyces species,* Abiotrophia *species*, Haemophilus *species*, Bacillus* species* *Fungal—Candida albicans,* Candida glabrata, Candida tropicalis, Candida krusei	[69, 71, 87, 143, 145–147]
Erythroplakia	Smokeless form: Betel (Areca) nut Smoking form: Cigarette smoke	Bacterial—Streptococcus anginosus, *Oribacterium*, *S*treptococcus *infantis, Actinomyces species* Fungal*—Candida albicans*	[69, 87]
Oral submucous fibrosis	Smokeless form: Betel (Areca) nut Smoking form: Cigarette smoke	Bacterial—Streptococcus anginosus, *Oribacterium*,* S*treptococcus *infantis, Actinomyces species* *Fungal—Candida albicans*	[69, 85]
Oral cancer	Smokeless form: Betel (Areca) nut, Khaini, Maras powder, ketone and amine components Smoking form: Cigarette smoke (nitrosamines)	Increase in (compared to normal mucosa): Bacterial*—Streptococcus anginosus, Porphyromonas gingivalis, Veillonella, Fusobacterium nucleatum, Prevotella intermedia, Actinomyces, Clostridium, Haemophilus parainfluenza, Enterococcus faecalis, Escherichia coli, Abiotrophia species, Streptococcus anginosus* *Fungal—Candida albicans, Candida glabrata*, *Candida tropicalis, Candida inconspicua*, *Candida famata, Candida kefyr, Saccharomyces cerevisiae, Candida krusei* Reduction in (compared to normal mucosa): Bacterial—*Streptococcus species, Staphylococcus species, Neisseria species, Peptostreptococcus species, Propionibacterium species, Capnocytophaga species, Firmicutes species and Actinobacteria species*	[71, 79, 80, 83, 86, 87, 98, 100, 147–151]
